# Damage Evaluation Based on a Wave Energy Flow Map Using Multiple PZT Sensors

**DOI:** 10.3390/s140201902

**Published:** 2014-01-23

**Authors:** Yaolu Liu, Ning Hu, Hong Xu, Weifeng Yuan, Cheng Yan, Yuan Li, Riu Goda, Alamusi, Jinhao Qiu, Huiming Ning, Liangke Wu

**Affiliations:** 1 Department of Mechanical Engineering, Chiba University, Chiba 263-8522, Japan; E-Mails: liuyaolu@graduate.chiba-u.jp (Y.L.); r.goda@chiba-u.jp (R.G.); alamusi@chiba-u.jp (A.); ninghuiming@gmail.com (H.N.); goraka@chiba-u.jp (L.W.); 2 Department of Engineering Mechanics, Chongqing University, Chongqing 400044, China; 3 Mechanical and Electrical Engineering Institute, Beijing University of Chemical Technology, 15 Beisanhuan Dong Lu, Chaoyang District, Beijing 100029, China; E-Mail: xuhong@mail.buct.edu.cn; 4 School of Manufacturing Science and Engineering, Southwest University of Science and Technology, Mianyang 621010, China; E-Mail: yuanweifeng@swust.edu.cn; 5 School of Chemistry, Physics and Mechanical Engineering, Queensland University of Technology, Brisbane QLD 4001, Australia; E-Mail: c2.yan@qut.edu.au; 6 Department of Nanomechanics, Tohoku University, Sendai 980-8579, Japan; E-Mail: liyuan@ism.mech.tohoku.ac.jp; 7 State Key Laboratory of Mechanics and Control of Mechanical Structures, Nanjing University of Aeronautics and Astronautics, Nanjing 210016, China; E-Mail: qiu@nuaa.edu.cn

**Keywords:** nondestructive inspection, ultrasonic waves, wave energy flow, signal processing, visualization technique

## Abstract

A new wave energy flow (WEF) map concept was proposed in this work. Based on it, an improved technique incorporating the laser scanning method and Betti's reciprocal theorem was developed to evaluate the shape and size of damage as well as to realize visualization of wave propagation. In this technique, a simple signal processing algorithm was proposed to construct the WEF map when waves propagate through an inspection region, and multiple lead zirconate titanate (PZT) sensors were employed to improve inspection reliability. Various damages in aluminum and carbon fiber reinforced plastic laminated plates were experimentally and numerically evaluated to validate this technique. The results show that it can effectively evaluate the shape and size of damage from wave field variations around the damage in the WEF map.

## 1. Introduction

In order to improve the safety, reliability and operation life of various aged structures, development of powerful nondestructive inspection techniques to detect possible defects is critical. To date, some techniques, e.g., X-ray inspection [1], infrared temperature measurement [2], thermography [3], eddy-current detection [4], Lamb wave tomography [5], ultrasonic C-scan [6], *etc.*, have been developed. Those methods based on ultrasonic waves [5–8] have been attracting increasing attention and ultrasonic scanning [6] is one of the most commonly used techniques in practice. With this method, a probe scanning at the surface of a structure generates bulk ultrasonic waves, which then propagate *along the thickness direction*. Internal structural defects can be evaluated by analyzing the time-domain or frequency-domain signal characteristics of transmitted or reflected waves caused by the defects. However, the inspection region of this technique is relatively small. In addition, overlapping and interference of multiple reflected and diffracted waves make the estimation a technical challenge as the validation of inspection results may largely depend on the experience and skill of inspectors. It is quite possible to overlook or even misinterpret some types of defects.

To deal with these problems, some new damage monitoring or inspection techniques based on Lamb waves propagating over a long distance in *structural span directions*, have been recently developed. The reliability of these approaches has been confirmed in the time reversal method [9–17] and probability-based imaging techniques [18]. The time reversal method is a powerful wave signal conversion technique, which is basically based on Betti's reciprocal theorem, and needs comparatively complex mathematical and experimental operations. The probability-based imaging approaches need base-line data of intact specimens, which is basically more suitable for on-line health monitoring compared to off-line evaluation. Based on the laser scanning method (LSM) and Betti's reciprocal theorem, Takatsubo *et al.* [19–21] proposed a simple visualization technique using ultrasonic Lamb wave propagation to perform damage inspections. In this method, ultrasonic elastic waves are thermally excited by a pulse laser in a scanned inspection region, and then collected using a fixed acoustic emission (AE) sensor on the surface of a test body. Based on Betti's reciprocal theorem, the waveform propagating from a laser irradiating point to the AE sensor can be directly converted into the waves originating from the sensor and then propagating to the laser irradiating point. Then, a series of snapshots of the wave propagation from the artificial wave source (the sensor position) to the scanned inspection region can be constructed. In this way, defects can be easily identified by directly observing wave scattering caused by them in the snapshots of the wave propagation at different time points, leading to high inspection reliability. Note that this method focuses on the *reciprocity of space*, *i.e.*, the locations of wave source (actuator) and sensor can be exchanged without any influence on wave propagation in linear and elastic problems. This idea is different from the traditional time reversal method that imitates *time back*. Moreover, compared with the conventional ultrasonic scanning methods, this technique has the following advantages: quick inspection of a large area; no adjustment on the incident angle of laser irradiation due to stable ultrasonic waves excited by thermal expansion, no need to move sensor, far field operation, simple signal processing, *etc*. Unfortunately, although the damage location can be easily identified with this method, often with high accuracy, damage shape and size cannot be evaluated.

In this work, we propose a new wave energy flow (WEF) map concept to evaluate the shape and size of damage areas. The technique proposed by Takatsubo *et al.* [19–21] was improved by using multiple cheap lead zirconate titanate (PZT) sensors instead of a single AE sensor to visualize wave propagation. To validate the improved technique including a new signal processing algorithm, an elliptical through hole or a non-penetrating slit in aluminum plates and invisible internal delamination in a carbon fiber reinforced plastic (CFRP) laminated plate were experimentally evaluated. In addition, numerical simulations were carried out to confirm the obtained experimental results.

This article is arranged as follows: the improved technique is described in detail in Section 2 and the experimental procedure is depicted in Section 3.1. The experimental and numerical investigations for various damages in aluminum plates and a CFRP laminated plate are reported in Sections 3.2 and 3.3. Finally, some conclusions are drawn in Section 4.

## 2. Technique Based on WEF Map

**Figure 1.**
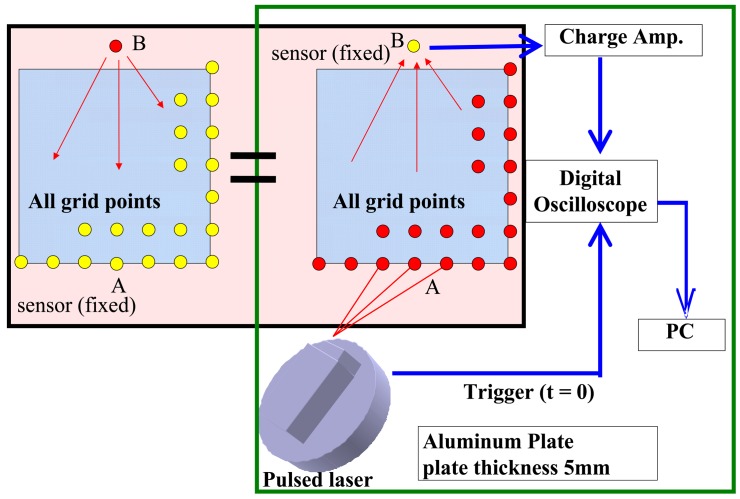
Schematic illustration of experimental setup.

As shown in Figure 1, the wave propagating from the laser scanning point A to the sensor B can be directly converted into that propagating from the sensor B to point A based on Betti's reciprocal theorem [19–21]. When irradiating all grid points in an inspection region using LSM, these data can be collected and transformed. With this new data set, the sensor works as an “artificial actuator” and the generated waves propagate from it toward the inspection region. Therefore, in the present work, “sensor” can be considered as “actuator” or wave source point.

The present improved technique based on WEF map is innovative and has two advantages compared with the previous technique [19–21]. First, a simple signal processing algorithm can be adopted to construct the WEF map. As we all know, elastic wave propagation in media can be understood as an energy propagation phenomenon, where the wave energy consisting of mutually interchanged elastic potential energy and kinetic energy is moving forward from a source point. The WEF map represents the distribution of a quantity, which is approximately equivalent to total wave strain energy density during an inspection time period when ultrasonic waves propagate through an inspection region. For a specified scanned point within the inspection region, this equivalent total wave strain energy density passing through this point during the inspection time period can be obtained by using a simple signal processing algorithm proposed in this work. Because the strain energy changes when waves propagate through damage or discontinuity, the detailed information about the damage, e.g., shape and size, can be simply evaluated from the WEF map. To construct the WEF map, the total wave strain energy density passing through all grid points in the inspection region within a sampling period should be estimated. Unlike the AE sensor used in [19–21], PZT sensors are employed here to collect wave signals, which represent the sum of two in-plane strain components, *i.e.*, *ε_x_* + *ε_y_*. Therefore, it is easy to estimate a quantity related to strain energy by using the PZT sensor signals directly. Note that the strain energy density can be expressed as: ${\left({ɛ}_{x}+{ɛ}_{y}\right)}^{2}(\lambda /2+G)+G(-2{ɛ}_{x}{ɛ}_{y}+\gamma_{xy}^{2}/2)$ for an isotropic elastic plane problem with two elastic Lame constants, *λ* and *G*. Therefore, the square of the PZT sensor signal is proportional to the first term in the above expression. Here, a quantity *γ* being approximately equivalent to the above strain energy density, which is named as *normalized strain energy density*, can be estimated by using the following Equation:

(1) $$\{\begin{array}{ll} \gamma =\overset{n}{\underset{i=1}{\text{∑}}}{\alpha_{i}}^{2} & \left(i=1,2,3,\cdots ,n\right)\\ n=T/\Delta T & \\ \alpha_{i}=\beta ({ɛ}_{xi}+{ɛ}_{yi}) & \end{array}$$

where *T* is the sampling time period when ultrasonic waves propagate through the inspection region, Δ*T* is the sampling interval, *β* is a proportion constant, *ε_xi_* is the strain in X direction at the *i*th sampling point within *T*, *ε_yi_* is the strain of Y direction. In experiments, the wave signal amplitude (unit: V) of a PZT sensor at the *i*th sampling point within *T* was used, on the assumption that it is proportional to the sum of the in-plane strains, *i.e.*, *ε_xi_* + *ε_yi_*. After applying Equation (1) to every grid point in the inspection region, the WEF map, denoting the distribution of *γ*, *i.e.*, total normalized strain energy density in the inspection region where ultrasonic waves propagate through during *T*, can be constructed. Unlike [19–21], which only use the sensor data at a specific time point to reproduce the wave propagation image at this time point, *γ* in Equation (1) is the sum of the squares of the strain signal data during *T*. Generally, as the strains of elastic ultrasonic waves are very small, the proportional constant *β* in Equation (1) was taken as 1,000 in this work.

Second, multiple PZT sensors with a suitable placement were used to avoid possibly missing some severe defects or erroneous recognition in damage detection. For instance, it was found that the intensity of the wave reflected from a crack is very weak when the angle between the incident wave direction and the crack length direction is small [19–22]. This problem cannot be solved when only two fixed sensors (two artificial actuators here) are used since the straight line connecting them may be parallel to the crack length direction. Therefore, by arranging three or more fixed sensors to form a network, strong interaction between incident waves and damage can be induced from at least one sensor, leading to high reliability of inspection. In this work, three sensors were placed in an equilateral triangle pattern around an inspection region, as shown later. In this case, even if the straight line connecting any two sensors is parallel to a crack length direction, missing of the crack can still be prevented by the third sensor.

## 3. Experimental and Numerical Procedures for Damage Evaluation

### 3.1. Experimental Set-Up

A schematic diagram of the experimental setup is shown in Figure 1. The following main devices and components were used: a pulse laser device (lamp-pumped pulse YAG laser, Brilliant Ultra Stable, Big Sky Laser Tech., Inc., Bozeman, MT, USA), three PZT sensors (C6, diameter 10 mm, thickness 0.5 mm, 200 kHz resonant frequency, FUJI Ceramics Co., Shizuoka, Japan), three charge amplifiers (DLPCA-200, FEMTO Inc., Carlisle, OH, USA), an oscilloscope (TDS3034B, Tektronix Inc., Tokyo, Japan) and a personal computer. Note that the duration of the laser pulse is 8.5 ns (120 MHz). For the present laser irradiation, due to the comparatively slow heating speed in the aluminum and CFRP specimens, the wave spectrum shows that the wave energy is mainly distributed from 50 kHz∼300 kHz depending on the specimens.

There are the following six steps in this experiment.

- Step 1. Determine the size of scanning interval. The scanning interval should be equal to or less than the half wavelength of Lamb waves propagating in the inspected structure to obtain a satisfactory result. In the aluminum and CFRP specimens, both S_0_ mode (large wavelength) and A_0_ mode (small wavelength) are generated by the pulse laser due to thermal expansion. The average wavelength of A_0_ mode within the above frequency domain is around 10 mm. Therefore, the inspection region was divided into a square grid pattern with an interval of 5 mm. This scanning interval is obtained from our experimental experience, which can yield the high-quality wave propagation images with the minimum scanning data. Too large scanning interval may lead to the low-quality wave propagation images or even discontinuous wave fronts.
- Step 2. Project the pulse laser to a grid point to excite Lamb waves.
- Step 3. Receive the response signals by the three PZT sensors which were firmly bonded on the specimen using epoxy resin adhesive.
- Step 4. Import the signals into the computer through the charge amplifier and the oscilloscope. The length of recorded wave signals should be sufficiently long to ensure that these waves can completely pass through the inspection region. For instance, the velocities of S_0_ mode and A_0_ mode in the present aluminum plate are about 5,000 m/s and 3,000 m/s, respectively, and they are of the similar, but slightly higher values in the CFRP plate. The dimensions of inspection region are 200 × 200 mm^2^ for the aluminum plates, and 100 × 100 mm^2^ for the CFRP plate. Therefore, the length of the recorded signals was approximately determined as 100 μs. Band-pass filtering processing was conducted on the receiving data to remove experimental noises.
- Step 5. Repeat the above Step 2–Step 4 to scan every grid point.
- Step 6. Process the recorded signals using Equation (1) to calculate *γ* at every grid point, and then use *γ* of all grid points to construct the WEF map. Note that in the present experiments, there may be mode changes between A_0_ and S_0_ modes at damage area (e.g., non-penetrating slit in the aluminum plate or delamination in the CFRP plate). However, these mode changes do not affect the present technique because the new algorithm adds up the signal amplitudes at all sampling points in an entire signal during *T* no matter what wave modes are contained in this signal (see Equation (1)).

### 3.2. Evaluation of Damage in Aluminum Plates

**Figure 2.**
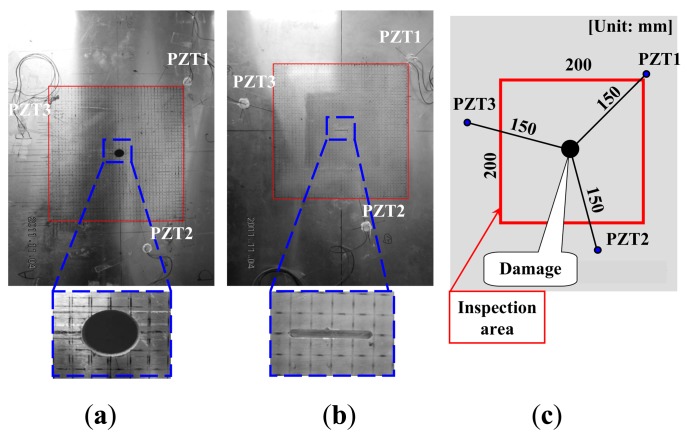
An aluminum plate: (**a**) with an elliptical through-hole; (**b**) with a non-penetrating slit; (**c**) position of sensors and damage.

At first, an aluminum plate with an elliptical through hole (minor-axis 12 mm, major-axis 15 mm) as shown in Figure 2a was used to experimentally and numerically validate the technique of WEF map. Although the elliptical through hole is obviously not a real damage in practical cases, it was used here for validation purpose only due to its simplicity. The thickness of the aluminum plate was 5 mm. The dimensions of inspection region were 200 × 200 mm^2^. Three PZT sensors were placed as shown in Figure 2c. Note that, for the aluminum plate, the spectrum of the present experimental Lamb waves mainly ranged from 100 kHz to 200 kHz, which were set as the lower and upper limits of signal band-pass filtering processing. Numerical simulations based on the pseudospectral Mindlin plate element proposed by the present authors [23] were also carried out to simulate wave propagation. Lamb waves were excited by applying the same wave signal as that generated by the pulse laser in the experiment to a PZT actuator. The computational time for one numerical simulation was around 4∼5 h when using a personal computer. The length of numerical wave signals used to construct WEF map was also 100 μs, which is the same as that used for experimental data.

**Figure 3.**
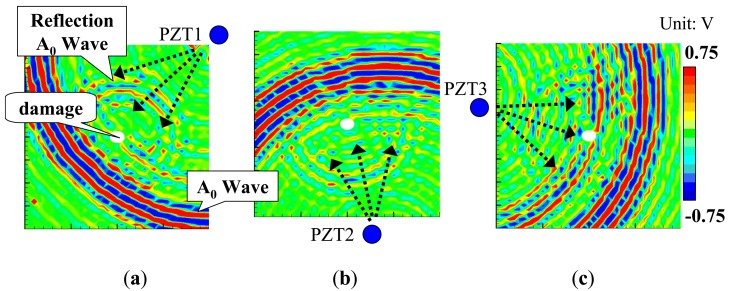
Experimental images of Lamb waves (time = 70 μs) using previous technique [19–21] for the aluminum plate with an elliptical through-hole: (**a**) PZT-1; (**b**) PZT-2; (**c**) PZT-3.

For comparison, at 70 μs, the snapshots of Lamb wave propagation from the PZTs 1, 2 and 3 obtained by the previous technique [19–21] are presented in Figure 3. By observing the wave scattering caused by the elliptical through hole in visualized wave propagation images, the position of the damage can be easily identified. However, the shape and size of damage area cannot be evaluated.

**Figure 4.**
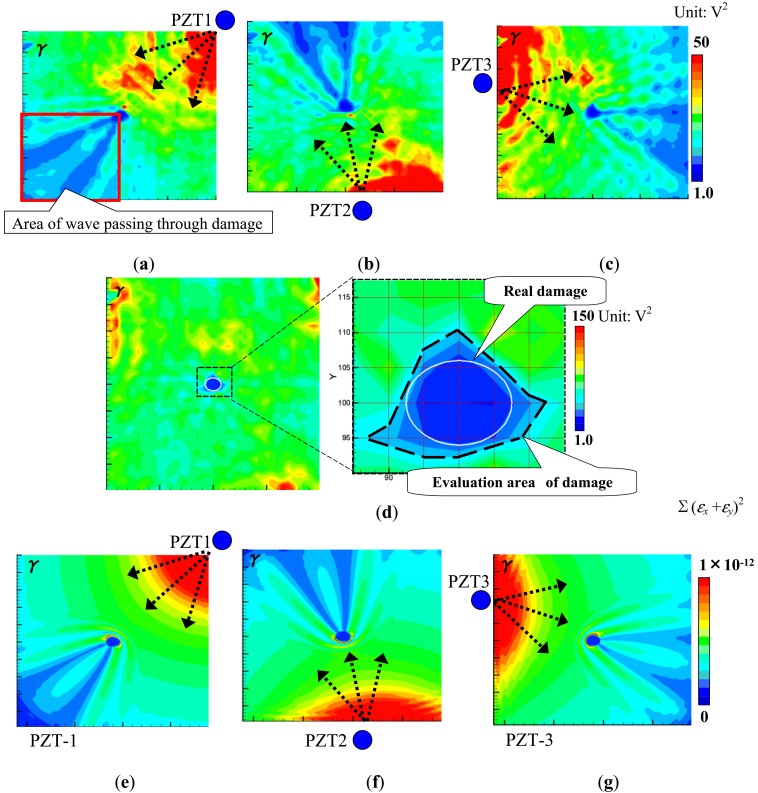
Images of *γ* distribution using the present WEF map technique for the aluminum plate with an elliptical through-hole: (**a**) experimental result of PZT-1; (**b**) experimental result of PZT-2; (**c**) experimental result of PZT-3; (**d**) experimental result of PZT-(1 + 2 + 3); (**e**) numerical result of PZT-1; (**f**) numerical result of PZT-2; (**g**) numerical result of PZT-3.

The results obtained by the present technique based on the WEF map are shown in Figure 4. There are two points in Figure 4a–c which should be highlighted. The first one is that the normalized strain energy density *γ* in the hole area is very low. This is because that there is no Lamb waves generated when the inside region of the hole is illuminated by the pulse laser. The second one is that the *γ* in the area behind the hole decreases significantly due to strong reflections at the hole. The WEF maps of the three PZTs are added up as shown in Figure 4d. It can be found that the damage shape and area can be evaluated accurately compared to the real damage. For comparison, the numerical results of *γ* = Σ(*ε_x_*+*ε_y_*)^2^ by setting *β* = 1 in Equation (1) are shown in Figure 4e–g. It can be identified that the numerical simulation results have the similar variation trends compared to the experimental ones, which indicates the validity of the present technique based on the WEF map.

**Figure 5.**
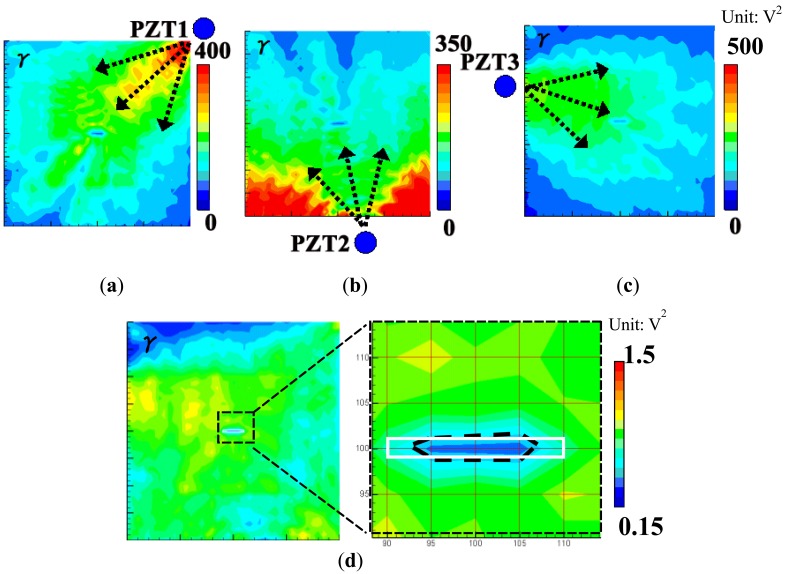
Experimental images of *γ* distribution for the aluminum plate with a non-penetrating slit (irradiated side with slit): (**a**) PZT-1; (**b**) PZT-2; (**c**) PZT-3; (**d**) PZT-(1 + 2 + 3).

Next, an aluminum plate with a non-penetrating slit (length: 20 mm, width: 2 mm and depth: 2.5 mm) as shown in Figure 2b was used to experimentally validate the present WEF map technique. Similar to the case of the through hole, the surface non-penetrating slit was used as damage due to its simplicity. The thickness of the aluminum plate was 5 mm. The dimensions of inspection region were 200 × 200 mm^2^. The laser scanning both on the side with the slit and the opposite side was carried out. Firstly, for the side with the slit, the experimental results are shown in Figure 5. The decrease of the normalized strain energy density *γ* in the slit area and that behind the slit caused by the reflection of waves can be clearly observed. Figure 5d shows the sum of *γ* using the three PZTs. It can be found that the experimentally evaluated damage (black dashed line) agrees with the real one well in shape and size. Note that the scanning points located in the slit region were also irradiated by the pulse laser.

**Figure 6.**
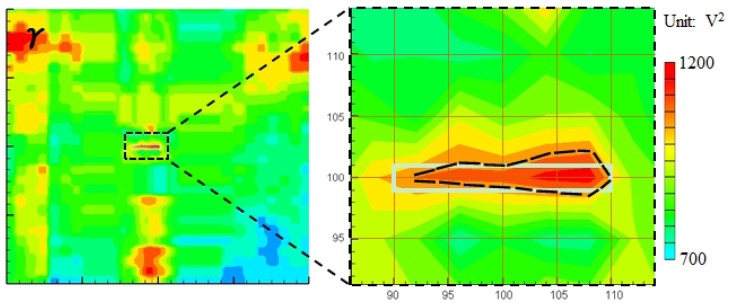
Experimental images of γ distribution for the aluminum plate with a non-penetrating slit (irradiated side without slit, result of PZT-(1 + 2 + 3)).

For the opposite side of the slit, it is useful to imitate some internal damages, such as an internal surface corrosion pit in a pipe because an inspector cannot find it from the inspected side using naked eyes. In this case, a similar result was obtained as shown in Figure 6. However, unlike Figure 5d, the normalized strain energy density *γ* in the slit area in Figure 6 is higher than those in the neighboring regions of the slit. The reason may be from the following two aspects. The first one is that, when waves pass through the slit, there should be no obvious reflections in the slit region since the irradiated surface is of no discontinuity. The second one is that, when waves pass through the slit, there should be some strain concentrations or higher strains in the slit region with the smaller thickness. Moreover, there are some misrecognized regions near plate boundaries in Figures 5 and 6, which should be caused by the strong reflections from the boundaries, *i.e.*, boundary effects, or the superposition of the reflections from the slit and those from the boundaries (e.g., the region near the bottom of the inspected area in Figure 6).

Finally, although satisfactory results are obtained in the present example when the slit is located on a grid line, it should be further justified in the future research that the relative position between grid (excitation) points and a damage would affect the results or not, that is, if the slit is not located on the grid points, the results would change or not.

### 3.3. Evaluation of Delamination in a CFRP Laminated Plate

**Figure 7.**
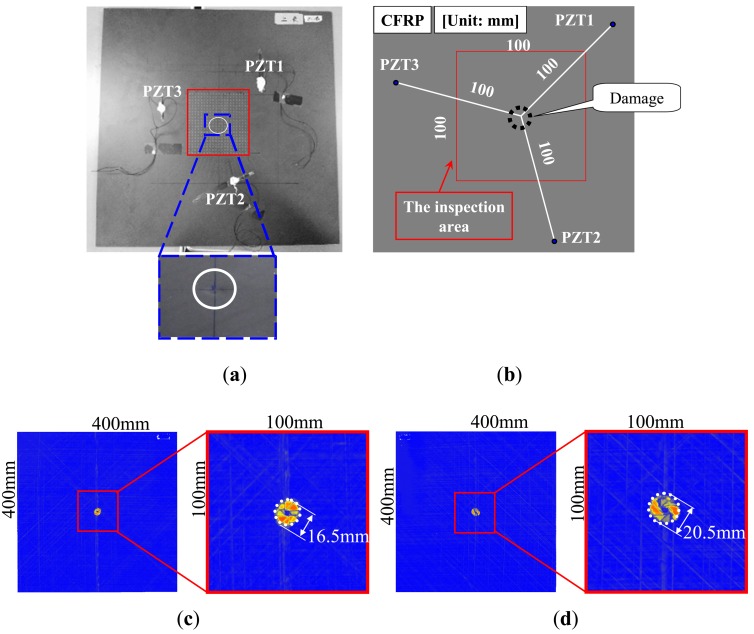
Test specimen (CFRP laminated plate): (**a**) delamination damage; (**b**) position of sensors and damage; (**c**) images of delamination at impacted side; (**d**) images of delamination at opposite side of impact.

A 32-layer quasi-isotropic CFRP laminated plate with a stacking configuration of [(45°/0°/−)45°/90°;_4_]_s_ as shown in Figure 7a was used to experimentally validate the present WEF map technique. The thickness of the CFRP laminated plate was 4.8 mm. For CFRP laminated plates, invisible internal delamination is one of the most dangerous damage patterns since it can significantly reduce the compressive strength of CFRP [24,25]. In this work, invisible internal delamination was induced by performing a low-velocity impact test using a Dynatup 9250HD weight-drop impact test machine (Instron Inc., Norwood, MA, USA). The center of the plate was impacted by an impacting body of a lower semi-spherical shape, the mass of 4.6 kg and impact energy of 7.0 J.

For reference, Figure 7c,d demonstrate the images of the invisible internal delamination using traditional ultrasonic C-scan by putting the test specimen in water. The scanning was performed from the two sides of the specimen using the scanning interval of 0.5 mm. Figure 7c,d show that the delamination size in the opposite side of impact is slightly larger than that of the impacted side. The maximum diameter of the delamination is around 20.5 mm.

**Figure 8.**
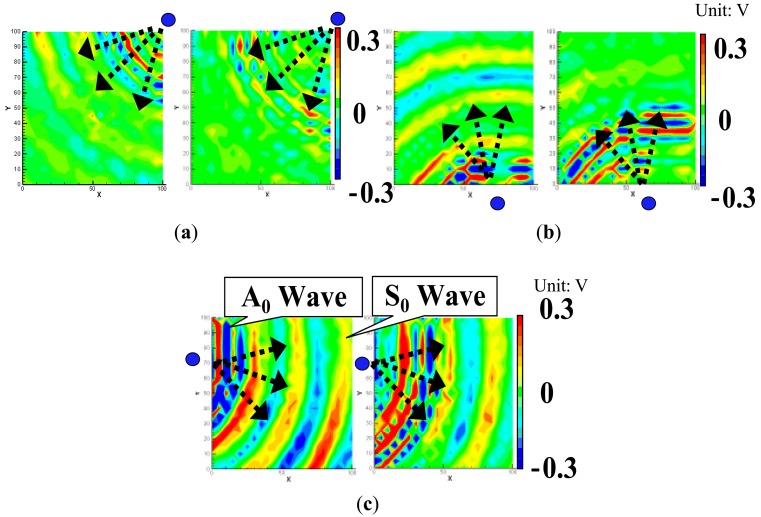
Experimental images of Lamb waves for CFRP laminate with delamination (time = 40 μs, 60 μs) using the previous technique [15–17]: (**a**) PZT-1; (**b**) PZT-2; (**c**) PZT-3.

As shown in Figure 7b, the impacted side of the CFRP laminated plate was inspected here with three PZT sensors attached, and the inspection region was 100 × 100 mm^2^. For the CFRP plate, the lower and upper limits of the band-pass filtering in frequency domain were set as 50 kHz and 300 kHz. For comparison, the snapshots of Lamb wave propagation from the PZTs 1, 2 and 3, obtained by the previous technique [19–21], are presented in Figure 8. The delamination cannot be easily identified since the wave scattering at the delamination is weak when using the present scanning grid interval of 5 mm. However, some researchers [19–21] have reported that the wave scattering in a delamination region could be observed by using the scanning grid interval of 1 mm.

**Figure 9.**
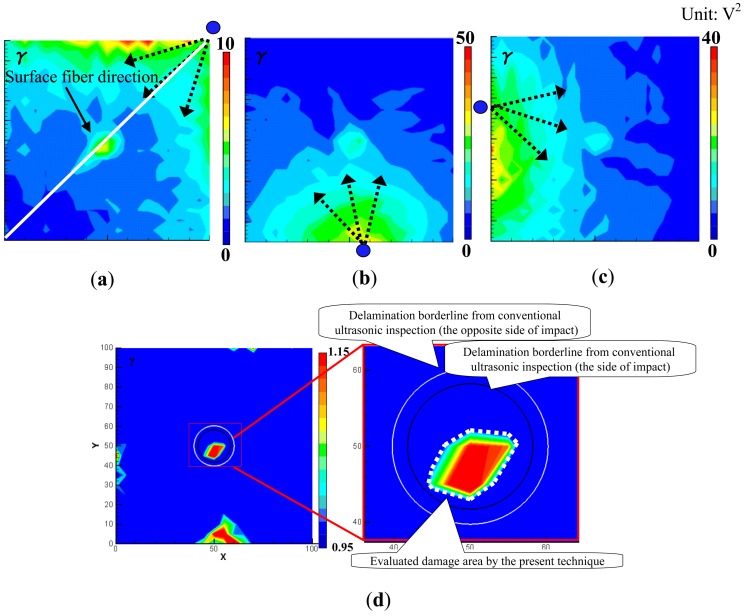
Experimental images of γ distribution using the present WEF map technique: (**a**) PZT-1; (**b**) PZT-2; (**c**) PZT-3; (**d**) PZT-(1 + 2 + 3).

Figure 9 shows the results obtained by the present WEF map technique. We can find that the *γ* in Figure 9a–c decreases after waves pass through the delamination area. However, compared to the previous results of the aluminum plates in Figures 4 and 5, this decrease is not so obvious. The reason may be that the incident waves can pass through the internal delamination more easily due to no surface discontinuity. Therefore, the reflected waves are relatively weaker, and consequently the influence of the delamination on the transmitted waves is small. Figure 9d shows the sum of the *γ* of the three PZTs. Unlike that in Figures 4 and 5, the *γ* increases in the delamination area in Figure 9d. The following two reasons may result in this phenomenon. One is that delamination generally leads to the bending stiffness reduction in the delamination area, and consequently bending strain in the delamination area should be larger compared to that of intact area. The other is that there may be many small transverse matrix cracks in the low-velocity impact-induced delamination region [26], leading to high strain concentration. Moreover, from Figure 9d, we can see that the position of the delamination can be identified accurately, but the evaluated delamination area (marked by a white dashed line) is slightly smaller than that obtained by the conventional ultrasonic C-scan. Compared to the interval of 0.5 mm used in the conventional ultrasonic scanning method, the present 5 mm grid interval may lead to the lower accuracy of damage evaluation.

**Figure 10.**
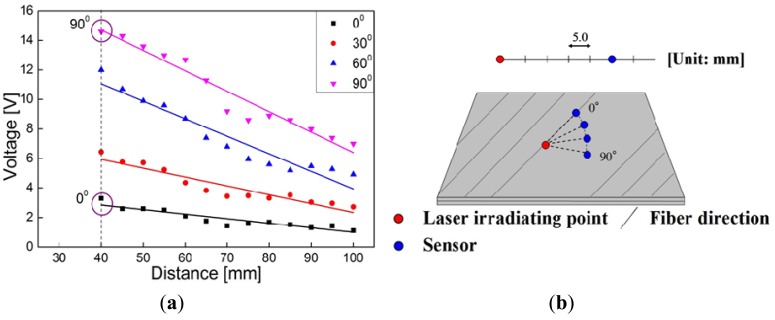
Amplitude of Lamb waves versus propagation distance (5 mm increment) and propagation direction (30° increment) in a CFRP plate: (**a**) relationship between distance of propagation and amplitude (fitted by least squares method of a linear relationship); (**b**) experimental model of the measurement.

In Figure 9d, there are some areas of high *γ*, especially at the bottom of the inspection region, which may result in false recognition. This phenomenon may be due to the material anisotropy of CFRP as discussed below. First, the maximum values of *γ* shown in Figure 9a–c are quite different. As shown in Figure 10, due to a much lower thermal expansion coefficient in the fiber direction, it was found that when an angle between the direction of wave propagation and the surface fiber orientation is closer to 0°, the initial amplitude of the waves becomes smaller when using the laser irradiation to generate Lamb waves [5]. In contrast, when the angle is closer to 90°, the initial amplitude of the waves becomes larger.

Therefore, by observing Figure 9a, the maximum value of *γ* is much lower than those in Figure 9b,c. In this case, the angle between the direction of wave propagation and the surface fiber orientation is very small. On the other hand, the maximum values of *γ* in Figure 9b,c are very high since the wave propagates almost vertically to the surface fiber direction, which cause the unreal damage areas located on the left and bottom boundaries of the inspection region. Second, the square operation of $\alpha_{i}^{2}$ in Equation (1) further amplifies the influence of material anisotropy.

**Figure 11.**
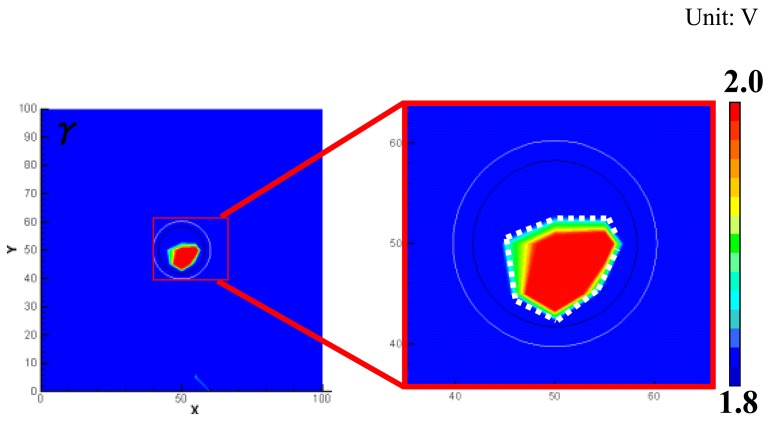
Damage image using absolute value of sensor signals in a CFRP plate with delamination.

To weaken this anisotropy influence, the sum of absolute value of the wave signals rather than the sum of squares in Equation (1) was used to construct the WEF map. The improved WEF map is shown in Figure 11. It can be seen that the unreal damages at the boundaries are removed and the evaluated damage area is slightly larger and more accurate than that in Figure 9d.

## 4. Conclusion

Based on the concept of WEF map, an improved technique incorporating LSM and Betti's reciprocal theorem was developed to evaluate the shape and size of damage, as well as to visualize wave propagation. In this technique, a simple signal processing algorithm was proposed to construct the WEF map, and multiple PZT sensors were employed to improve inspection reliability and to reduce cost. Two kinds of damages in aluminum plates and invisible internal delamination in a CFRP laminated plate were used to experimentally validate the proposed technique. In addition, numerical simulations were carried out to confirm the experimental results. The conclusions are summarized as follows:

(1) The damage position in both aluminum and CFRP laminated plates can be accurately identified by the present improved technique based on the WEF map.
(2) For aluminum plates with an elliptical through hole or a non-penetrating slit, the damage shape and size can be evaluated with comparatively high accuracy. For the CFRP laminated plate with internal delamination, the evaluated delamination area is slightly smaller than the real one. Moreover, it is important to weaken the effect of material anisotropy to improve the quality of delamination image.
(3) There are still some weaknesses in the present technique, leading to instances of inspection uncertainty or misrecognition. Because the present technique is based on the information of strain within a selected time period, besides damages, a lot of other factors can cause the variation of strain, e.g., scattering, propagation distance, boundary effects, stiffeners in complex structures, *etc*. For instance, wave scattering in various directions due to localized non-uniformities in medium may influence the identification accuracy of damage size and shape, strong boundary reflections may lead to some misrecognized regions, and stiffeners with perfect bonding to host structures may also cause the variation of strain value. All of these issues will be addressed in our future work.
